# Validation of the Shiraz Verbal Learning Test in elderly women, both with and without dementia

**DOI:** 10.1590/1980-5764-DN-2025-0428

**Published:** 2026-10-12

**Authors:** Afsaneh Haghdel, Hassan Haghshenas, Sahar Zabihidan

**Affiliations:** 1 Apadana Institute of Higher Education, Faculty of Humanities, Department of Psychology – Shiraz, Iran.

**Keywords:** Memory and Learning Tests, Dementia, Episodic Memory, Neurocognitive Disorders, Aged, Testes de Memória e Aprendizagem, Demência, Memória Episódica, Transtornos Neurocognitivos, Idosas

## Abstract

Dementia is a major public health concern, and culturally adapted cognitive tools are essential for accurate screening among older adults.

**Objective:**

To evaluate the discriminant validity of the Shiraz Verbal Learning Test (SVLT) in differentiating verbal memory performance among elderly women with and without dementia in Shiraz, Iran.

**Methods:**

A cross-sectional study was conducted with 60 women aged 65 years or older, recruited between 2024 and 2025 by convenience sampling in Shiraz. The sample included 30 cognitively healthy participants and 30 with mild to moderate dementia diagnosed according to Diagnostic and Statistical Manual of Mental Disorders, Fifth Edition (DSM-5) criteria and confirmed by a neurologist. Assessments included a demographic questionnaire, the Mini-Mental State Examination (MMSE), the 28-item General Health Questionnaire (GHQ-28), and the SVLT. Data were analyzed using multivariate analysis of variance (MANOVA) and univariate analysis of variance (ANOVA).

**Results:**

The dementia group scored significantly lower than the healthy group across all SVLT memory measures — learning, immediate recall, delayed free recall, delayed cued recall, and recognition (p<0.01) — demonstrating strong discriminant capacity.

**Conclusion:**

The SVLT demonstrated satisfactory psychometric validity and promising clinical usefulness in differentiating dementia-related verbal memory deficits among elderly Iranian women, indicating its potential as a culturally adapted tool for early cognitive screening in clinical and research contexts.

## RESUMO

A demência é uma importante questão de saúde pública, e instrumentos cognitivos adaptados culturalmente são essenciais para uma triagem precisa em populações idosas.

**Objetivo:**

Avaliar a validade discriminante do Teste de Aprendizagem Verbal de Shiraz (SVLT) na diferenciação do desempenho de memória verbal entre mulheres idosas com e sem demência em Shiraz, Irã.

**Métodos:**

Este estudo transversal foi realizado com 60 mulheres de 65 anos ou mais, recrutadas entre 2024 e 2025 por amostragem por conveniência em Shiraz. A amostra incluiu 30 participantes cognitivamente saudáveis e 30 com demência leve a moderada, diagnosticada segundo os critérios do Manual Diagnóstico e Estatístico de Transtornos Mentais, 5ª edição (DSM-5) e confirmada por um neurologista. Foram aplicados questionário sociodemográfico, Mini-Exame do Estado Mental (MEEM), Questionário de Saúde Geral – 28 itens (QSG-28) e o SVLT. Os dados foram analisados por meio de análise multivariada de variância (MANOVA) e análise univariada de variância (ANOVA).

**Resultados:**

O grupo com demência apresentou escores significativamente inferiores aos do grupo saudável em todas as medidas de memória do SVLT — aprendizagem, recordação imediata, recordação livre tardia, recordação tardia com pistas e reconhecimento (p<0,01) — indicando forte capacidade discriminativa do instrumento.

**Conclusão:**

O SVLT demonstrou validade psicométrica satisfatória e utilidade clínica promissora para distinguir déficits de memória verbal associados à demência entre mulheres idosas iranianas, indicando seu potencial como instrumento culturalmente adaptado para triagem cognitiva precoce em contextos clínicos e de pesquisa.

## INTRODUCTION

Dementia is a major global public health challenge of the twenty-first century, defined as a neurocognitive syndrome marked by progressive decline in cognitive abilities and impaired daily functioning^1,2^. Approximately 50–55 million people worldwide are currently affected, with numbers projected to rise sharply due to population ageing^3,4,5^. Alzheimer’s disease is the most common type, followed by vascular, Lewy body, and frontotemporal dementias^2^. Beyond individual suffering, dementia imposes heavy economic and psychological burdens on families and healthcare systems^3^.

Iran is undergoing a demographic shift toward an ageing population, raising serious concerns about dementia prevalence^6^. The incidence was expected to reach 118 per 100,000 women and 109 per 100,000 men by 2024^7^, signaling an urgent need to expand medical and specialized care services^8^.

Verbal memory impairment is among the earliest and most prominent symptoms of dementia and crucial for early diagnosis^9^. Neuroimaging findings reveal that verbal memory deficits in Alzheimer’s disease are linked to specific neural networks^10,11^, with consistent recall-pattern disturbances compared with healthy controls^9^.

However, cognitive tests developed in Western contexts face significant challenges in non-English-speaking and culturally diverse groups, as they reflect Western linguistic and cultural norms^12^. Persian-speaking older adults often encounter linguistic and cultural barriers in neuropsychological assessments^13^. The scarcity of standardized Persian instruments underscores the need for culturally adapted tools^14^.

To address this gap, the Shiraz Verbal Learning Test (SVLT) was developed as a culturally relevant verbal memory measure for Persian speakers^15^. Although clinically promising^16^, further psychometric evaluation — particularly discriminant validity in clinical samples — is required. Discriminant validity reflects an instrument’s ability to distinguish between distinct populations^17^ and determines whether it can accurately differentiate people with dementia from healthy individuals^18^.

This study aimed to assess the discriminant validity of the SVLT in distinguishing cognitively healthy elderly women from those with dementia, as women have a longer life expectancy and a higher risk of developing dementia^19^. The SVLT evaluates memory performance across multiple trials and qualitative error indices, including intrusions and false recognitions^16^.

The focus on elderly women was intentional and guided by epidemiological, methodological, and sociocultural considerations. Methodologically, restricting the sample to women minimized gender-related variability in verbal memory performance and strengthened internal validity. Within the Iranian sociocultural context, norms governing gender interactions in clinical and community settings can influence participant accessibility and engagement during extended face-to-face neuropsychological assessments; accordingly, a single-sex study design enabled more feasible, consistent, and methodologically robust data collection.

Recent Iranian studies highlight the importance of cognitive screening for early detection and intervention^20^. Early diagnosis allows the implementation of targeted cognitive programs and reduces the social and economic burden^21,22^. Although brief tools such as the Mini-Mental State Examination (MMSE) and the Montreal Cognitive Assessment (MoCA) are widely used, they inadequately capture domain-specific memory deficits^23^. More comprehensive instruments, such as verbal learning tests, may thus offer greater diagnostic precision.

Despite the development of the SVLT, few studies have thoroughly examined its discriminative potential between dementia and healthy aging. Such work would strengthen its clinical and research applicability and contribute to cross-cultural validation of verbal memory assessment tools.

## METHODS

### Study design and participants

A cross-sectional study was carried out in Shiraz, Iran (2024–2025) to evaluate the discriminant validity of the SVLT among elderly women with and without dementia. Sixty women aged ≥65 years were recruited through healthcare centers and nursing homes using convenience sampling. Participants were divided equally into two groups:

- those diagnosed with mildtomoderate dementia according to DSM5 criteria and confirmed by a neurologist (n=30); and
- cognitively healthy controls with no history of neurological or psychiatric disorders (n=30).

Sample size was based on previous studies, ensuring 80% power and α=0.05. Inclusion criteria were female gender, basic literacy, and informed consent. Exclusion criteria comprised major psychiatric disorders, other neurological diseases, traumatic brain injury, severe sensory impairment, or abnormal 28-item General Health Questionnaire (GHQ28) scores indicating psychological distress.

Dementia diagnoses were established by an experienced neurologist according to DSM5 criteria for Major Neurocognitive Disorder, based on clinical history, functional impairment, and neurological examination. The terms *“*mild*”* and *“*moderate*”* dementia were used solely as clinical severity descriptors within the Major Neurocognitive Disorder framework and do not represent formal DSM5 diagnostic categories.

Cognitive severity was operationalized using global cognitive performance. The MMSE was applied as a standardized screening and severity-characterization tool, not as a diagnostic instrument. MMSE scores were used to support group classification, confirm cognitive impairment in the dementia group, and exclude cognitively borderline cases. Cognitively healthy participants met age- and education-adjusted normative MMSE cutoffs indicative of preserved global cognition.

Ethical approval was obtained from the Research Ethics Committee of Shiraz University (IR.US.PSYEDU.REC.1403.083), and all participants or legal caregivers provided written informed consent.

### Instruments

#### Demographic questionnaire

A structured demographic questionnaire compiled by the researchers collected data on age, years of education, marital and occupational status, medical history, use of neuroactive medications, family history of dementia or Alzheimer’s disease, substance use pattern, body mass index (BMI), and living arrangements. Age and educational level were also used for group matching and to control potential confounding variables.

#### Mini-Mental State Examination (MMSE)

The MMSE is a 30-item screening test for global cognitive functioning, widely used in dementia research^24^. It evaluates five major domains: orientation (10 points), registration (3 points), attention and calculation (5 points), recall (3 points), and language and visuospatial abilities (9 points). Scores range 0–30, with ≥25 indicating normal cognition, 19–24 mild to moderate impairment, and <19 severe impairment. The Persian adaptation has demonstrated strong psychometric indices (test–retest r=0.87; inter-rater r=0.83)^25^. In this study, MMSE scores were used to confirm diagnostic grouping and exclude cognitively borderline cases.

The MMSE was administered to participants in the dementia group to support the clinical diagnosis, characterize the level of global cognitive impairment (mild to moderate), and exclude borderline or atypical cases. Cognitively healthy participants were screened based on the absence of neurological or psychiatric disorders, normal daily functioning, and GHQ-28 scores below the clinical threshold. Accordingly, MMSE data were considered methodologically meaningful only within the dementia group and were not used as a comparative measure between groups.

In the present study, the MMSE was used to support severity characterization rather than to establish DSM5 diagnoses. It functioned as a screening and inclusion/exclusion measure to ensure clear cognitive separation between groups.

The MMSE was administered in the dementia group to characterize global cognitive impairment after dementia had been clinically diagnosed by a neurologist according to DSM-5 criteria for Major Neurocognitive Disorder. Accordingly, MMSE scores served as a descriptive indicator of cognitive status rather than a diagnostic criterion. MMSE data were not obtained for the control group, as cognitively healthy participants were screened through clinical history, preserved functional status, and absence of significant psychological distress. Therefore, MMSE performance was not intended for direct between-group comparison; the study instead examined the discriminant validity of the SVLT across clinically defined groups.

#### General Health Questionnaire–28 (GHQ-28)

The GHQ-28 screens for psychological distress^26^ and comprises four seven-item subscales assessing somatic symptoms, anxiety–insomnia, social dysfunction, and depression. Items are rated on a 4-point Likert scale (0–3), providing a total score range 0–84. A cutoff of 23/24 differentiates probable cases of psychological disorder, with higher scores indicating greater severity. Reliability studies in Iranian samples report Cronbach’s α=0.85–0.93^27^. In the present study, participants scoring above the clinical threshold were excluded to minimize confounding due to psychological morbidity.

#### Shiraz Verbal Learning Test (SVLT)

The SVLT is a culturally adapted Persian variant of the California Verbal Learning Test (CVLT)^28^, standardized by Rahmani et al. (2017)^10^ for Persian-speaking populations^10^. It comprises a 16-word list derived from high-frequency nouns in Persian, read aloud over five consecutive learning trials, each followed by immediate free recall (Trials 1–5). After a second interference list, participants complete short-delay free recall, short-delay cued recall, long-delay free recall (20 minutes later), and a recognition trial comprising target and distractor words.

Beyond quantitative indices, the SVLT enables qualitative error analyses — intrusions, repetitions, and false recognitions — providing a comprehensive neurocognitive profile of verbal memory functioning. The Iranian standardization study reported satisfactory reliability (r=0.81) and internal consistency (Cronbach’s α=0.87)^10^. All core and error indices were analyzed to assess discriminant validity between dementia and healthy groups.

### Procedures

Participants meeting eligibility criteria were recruited following approval by the Research Ethics Committee of Shiraz University (IR.US.PSYEDU.REC.1403.083) and authorization from local care facilities. After a detailed explanation of the study aims, procedures, and rights, written informed consent was obtained from participants or legal caregivers.

Testing was conducted individually in quiet, welllit rooms at memory clinics, nursing homes, and elderly community centers. Evaluations lasted approximately 60 minutes per participant. The standardized battery was administered by a clinical psychologist trained in geriatric neuropsychological assessment.

For cognitively healthy controls, testing involved sequential administration of the demographic questionnaire and GHQ28 (≈15 min), followed by the SVLT (≈35–40 min). For participants with dementia, the MMSE (≈10 min) was given prior to the SVLT to verify diagnostic status. Examiner instructions were delivered verbatim as outlined in the test manuals to ensure procedural consistency.

Rest pauses were permitted when participants showed fatigue, and environmental distractions were minimized. All raw data were doublechecked and coded anonymously for statistical analysis.

### Ethical considerations

This research complied fully with the ethical standards set forth in the Declaration of Helsinki and was approved by the Research Ethics Committee of Shiraz University (approval code: IR.US.PSYEDU.REC.1403.083). All participants or their legal caregivers provided written informed consent after receiving explanatory information about the study’s aims and procedures. The confidentiality of all data was strictly maintained — personal identifiers were removed, and coded files were accessible only to authorized researchers. Participation was voluntary, with participants’ right to withdraw at any stage respected. The collected information was used exclusively for scientific purposes dedicated to advancing academic and clinical understanding of cognitive health in older adults.

### Statistical analysis

Data were processed using descriptive and inferential statistics. Means, standard deviations, and frequency distributions summarized participant characteristics and cognitive scores. Group comparisons for categorical and continuous variables were examined using χ^2^ tests and independent *t*-tests. To evaluate the discriminant validity of the SVLT, univariate analysis of variance (ANOVA) and multivariate analysis of variance (MANOVA) were conducted across all SVLT indices. Effect sizes (partial η^2^) and statistical power (1–β) were reported to quantify the magnitude and reliability of group differences. Statistical significance was set at p<0.05 for all tests. Analyses were performed using the Statistical Package for the Social Sciences (SPSS) version 27.0 (IBM Corp., Armonk, NY, USA).

## RESULTS

### Demographic characteristics

As summarized in Table 1, the two groups were comparable in age and education level. The mean age was 68.37 years (standard deviation ±3.24) in the cognitively healthy group and 68.47±3.13 years in the dementia group. Independent *t*-tests showed no significant differences in age (t(58) = –0.121; p=0.904) or years of education (t(58) = –0.322; p=0.749). Other demographic variables — including marital and occupational status — did not differ significantly (p>0.05).

**Table 1:** Demographic characteristics of participants (n=60).

Variable	Healthy group	Dementia group	Total sample (%)	Test	Test statistic	p-value	Result
Age (years)	M=68.37 SD=3.24	M=68.47 SD=3.13	M=68.42 SD=3.16	Independent *t*-test	t(58)= –0.121	0.904	Not significant
Years of formal education	M=10.37 SD=5.51	M = 9.93 SD=4.93	M=10.15 SD=5.22	Independent *t*-test	t(58)= –0.322	0.749	Not significant
Education level		—	Below high school: 33 (55.0) High school diploma: 12 (20.0) Bachelor’s: 8 (13.3) Master’s: 5 (8.3) PhD: 2 (3.3)	—	—	—	Descriptive only
Marital status		—	Single: 1 (1.7) Married: 49 (81.7) Divorced: 3 (5.0) Widowed: 7 (11.7)	—	—	—	Descriptive only
Occupational status		—	Housewife: 37 (61.7) Retired: 17 (28.3) Self-employed: 5 (8.3) Employee: 1 (1.7)	—	—	—	Descriptive only

Abbreviation: M, Mean age; SD, Standard deviation.

Notes: Data are presented as mean (standard deviation) or n (%); p-values were derived from independent *t*-tests. There were no statistically significant differences between groups in age, years of education, or other demographic variables (p>0.05).

### Global cognitive status (MMSE)

Within the dementia group, MMSE scores ranged from mild to moderate cognitive impairment. After excluding two cognitively healthy individuals who had undergone MMSE testing solely for initial screening purposes, the final dementia sample demonstrated a mean MMSE score of 23.25±2.54. This distribution reflects a relatively homogeneous level of global cognitive decline appropriate for examining associations between MMSE and domain-specific memory measures.

### Performance on the Shiraz Verbal Learning Test (SVLT)

Table 2 summarizes the results of the ANOVA for SVLT indices. The MANOVA revealed a significant overall group effect on SVLT performance (p<0.05). Followup univariate analyses indicated that cognitively healthy older women scored significantly higher than those with dementia across all learning, recall, and recognition measures.

**Table 2:** Results of univariate analysis of variance on the main indices of the Shiraz Verbal Learning Test.

Index	Healthy group (mean±SD)	Dementia group (mean±SD)	F	Sig. (p)	Partial η^2^	Power (1–β)
Trial 1	6.20±1.45	4.90±2.09	7,842	0.007	0.119	0.786
Trial 2	7.80±1.86	6.10±2.25	10,159	0.002	0.149	0.880
Trial 3	9.20±2.34	7.07±2.35	12,425	0.001	0.176	0.934
Trial 4	10.27±2.12	7.70±2.04	22,910	<0.001	0.283	0.997
Trial 5	11.00±2.15	8.50±2.49	17,344	<0.001	0.230	0.984
Immediate recall	6.30±1.82	4.63±2.15	10,450	0.002	0.153	0.889
Short-delay free recall	9.00±2.94	7.17±3.10	5,536	0.022	0.087	0.638
Short-delay cued recall	10.77±2.94	8.43±2.56	10,747	0.002	0.156	0.897
Long-delay free recall	9.63±2.65	6.73±2.92	16,229	<0.001	0.219	0.977
Long-delay cued recall	11.13±3.16	8.40±2.36	14,423	<0.001	0.199	0.962
Recognition	14.43±1.94	13.17±2.67	4,424	0.040	0.071	0.543

Abbreviation: SD, Standard deviation.

Note: Data are expressed as mean±standard deviation. Analysis of variance (F) results indicate significant group differences in most Shiraz Verbal Learning Test indices (p<0.05). The relatively high F values, medium-to-large effect sizes (partial η^2^), and satisfactory statistical power support the SVLT’s strong discriminative validity and reliability in detecting verbal memory deficits among older women with and without dementia.

### Immediate recall (Learning Trials and List B)

According to Table 3, significant betweengroup differences were found across all five learning trials and List B immediate recall (F:7,842–17,344; p<0.01). The healthy group consistently outperformed the dementia group on all immediate learning indices, with mediumtolarge effect sizes (η^2^: 0.12–0.23) and strong statistical power (1–β>0.78).

**Table 3:** Results of immediate recall analysis in the Shiraz Verbal Learning Test.

Index	Healthy group (mean±SD)	Dementia group (mean±SD)	F	p-value	Partial η^2^	Power
Trial 1	6.20±1.45	4.90±2.09	7.842	0.007	0.119	0.786
Trial 2	7.80±1.86	6.10±2.25	10.159	0.002	0.149	0.880
Trial 3	9.20±2.34	7.07±2.35	12.425	0.001	0.176	0.934
Trial 4	10.27±2.12	7.70±2.04	22.910	0.000	0.283	0.997
Trial 5	11.00±2.15	8.50±2.49	17.344	0.000	0.230	0.984
List B immediate recall	6.30±1.82	4.63±2.15	10.450	0.002	0.153	0.889

Abbreviation: SD, Standard deviation.

Note: Data are expressed as mean ± standard deviation (SD). F, analysis of variance statistic; p-value, statistical significance; Partial η^2^, partial eta-squared (effect size); Power, statistical power of the test. Statistical significance was set at p<0.05.

### Short-delay recall

Table 2 shows significant group differences for both free (F=5,536; p=0.022; η^2^=0.087; power=0.64) and cued (F=10,747; p=0.002; η^2^=0.156; power=0.90) conditions, reflecting moderate discriminant power of the SVLT in shortdelay memory retention.

### Longdelay recall

As reported in Table 2, longdelay free recall (F=16,229; p<0.001; η^2^=0.219; power=0.98) and cued recall (F=14,423; p<0.001; η^2^=0.199; power=0.96) showed the strongest group effects, confirming the SVLT’s sensitivity for delayed memory retrieval.

### Recognition

Table 2 also displays results for recognition performance, which was significantly higher in the healthy group (F=4,424; p=0.040; η^2^=0.071; power=0.54). Although the effect size was smaller than that observed in recall conditions, it still demonstrated the SVLT’s discriminative validity for recognition ability.

### Memory error analysis

Table 4 compares the mean frequency of memory errors between groups. Most indices revealed no significant differences. The only significant effect was observed for irrelevant responses during longdelay cued recall (F=4,798; p=0.033; η^2^=0.076; power=0.58), indicating greater cognitive disorganization and reduced inhibitory control among participants with dementia after prolonged retention intervals.

**Table 4:** Comparison of memory error indices in the Shiraz Verbal Learning Test between groups.

Error Index/Irrelevant Response	Healthy Group (mean±SD)	Dementia Group (mean±SD)	F	p-value	Partial η^2^	Power
Total repetitive responses (whole test)	3.40±2.90	2.30±2.57	2.417	0.125	0.040	0.333
Irrelevant responses in Trial 1	0.63±1.00	0.60±0.89	0.019	0.892	0.000	0.052
Irrelevant responses in immediate recall	0.70±0.84	1.00±1.36	1.054	0.309	0.018	0.172
Repetitive responses in immediate recall	0.37±0.93	0.37±1.65	0.000	1.000	0.000	0.050
Irrelevant responses in short-delay free recall	1.40±1.83	1.07±1.97	0.528	0.470	0.009	0.110
Repetitive responses in short-delay free recall	0.30±0.50	0.47±0.52	0.938	0.337	0.016	0.159
Repetitive responses in short-delay cued recall	0.03±0.19	0.03±1.40	0.000	1.000	0.000	0.050
Irrelevant responses in short-delay cued recall	2.07±2.87	2.57±2.86	0.866	0.356	0.015	0.150
Repetitive responses in long-delay free recall	0.60±0.76	0.57±0.86	0.018	0.893	0.000	0.052
Irrelevant responses in long-delay free recall	1.23±1.73	1.60±2.29	0.844	0.362	0.014	0.147
Repetitive responses in long-delay cued recall	0.00±0.00	0.00±0.00	n/a	n/a	n/a	n/a
Irrelevant responses in long-delay cued recall	**1.67±2.48**	**2.67±2.55**	**4.798**	**0.033**	**0.076**	**0.577**
Extra responses in recognition stage	5.57±1.24	6.70±2.08	0.950	0.334	0.016	0.160

Abbreviation: SD, Standard deviation.

Note: Data are expressed as mean±standard deviation (SD). F, analysis of variance statistic; p-value, statistical significance; Partial η^2^, partial eta-squared (effect size); Power, statistical power of the test (1−β); n/a, not applicable. Statistical significance was set at p<0.05.

### Associations between Mini-Mental State Examination and Shiraz Verbal Learning Test performance

To examine the relationship between general cognitive performance as assessed by the MMSE and verbal memory outcomes, Pearson correlation analyses were conducted between MMSE scores and key SVLT indices within the dementia group (n=30). MMSE scores showed significant positive correlations with SVLT Trial 5 learning performance (r=0.44; p=0.016), short-delay free recall (r=0.43; p=0.017), and long-delay free recall (r=0.42; p=0.022), indicating that higher MMSE scores were associated with better learning efficiency and free recall performance.

The correlation between MMSE scores and immediate recall did not reach statistical significance (r=0.28; p=0.136). Recognition performance showed a strong and statistically significant association with MMSE scores (r=0.57; p<0.001), suggesting that recognition accuracy increased in parallel with overall cognitive performance as indexed by the MMSE. This relationship is illustrated in Figure 1, which shows a clear positive linear trend between general cognitive performance, as assessed by the MMSE, and SVLT recognition ability.

**Figure 1:**
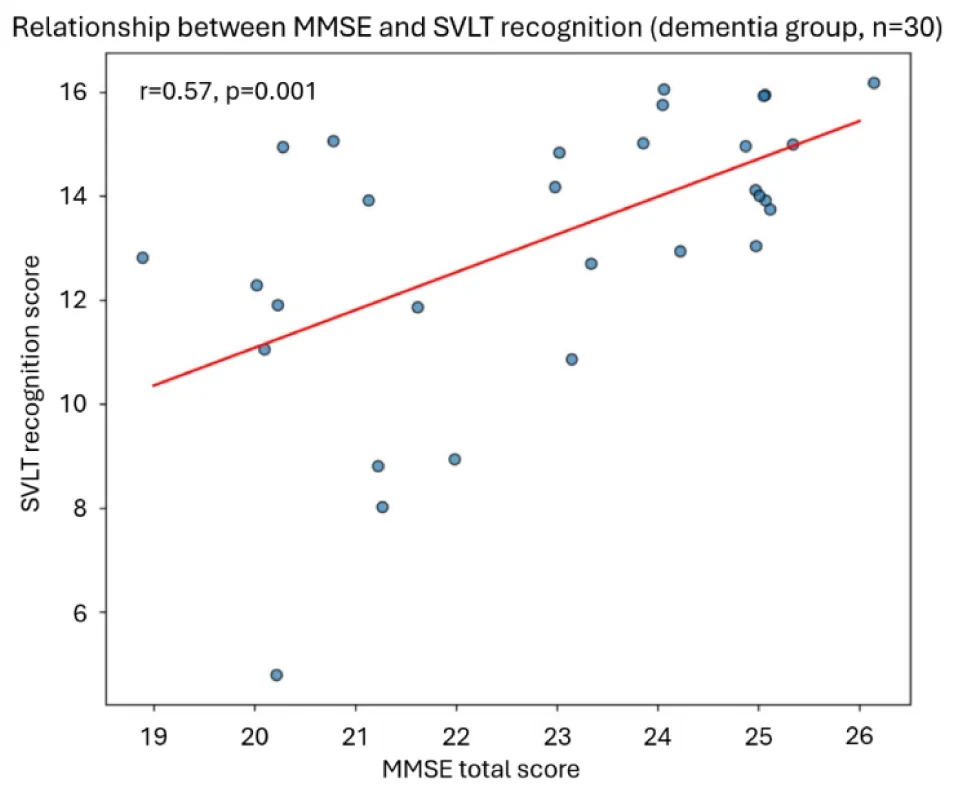
Scatter plot illustrating the relationship between Mini-Mental State Examination total scores and Shiraz Verbal Learning Tests recognition performance in the dementia group (n=30).

Notes: Each point represents one participant; slight jitter was applied to improve visibility due to overlapping scores. The solid line represents the fitted linear regression, demonstrating a significant positive association between MMSE scores and SVLT recognition performance (r=0.57; p<0.001).

## DISCUSSION

The findings of the present study indicated that the SVLT demonstrated a strong discriminative capacity in differentiating verbal memory performance between older women with dementia and cognitively healthy controls. The dementia group obtained lower scores across all learning trials (Trials 1–5), immediate recall, short- and long-delay recall (both free and cued), as well as recognition. The F values ranged from 4.42 to 22.91, with partial η^2^ values between 0.07 and 0.28, reflecting the satisfactory sensitivity of SVLT indices in distinguishing memory functions associated with dementia. These differences, together with mediumtolarge effect sizes and adequate statistical power, support the discriminant validity of the SVLT for detecting dementiarelated memory impairment.

The significant reductions in delayed recall (both free and cued) and recognition are consistent with Alzheimer’s disease models and other dementias involving hippocampal and medial temporal lobe dysfunctions, which play critical roles in episodic memory encoding and consolidation. Damage to these regions results in reduced retention over delay intervals, a phenomenon highlighted in recent biological–clinical frameworks of dementia^29,30,31^. The more pronounced deficits in delayed components and in certain learning trials (particularly Trials 4 and 5, which showed larger effect sizes) align with the “encoding/consolidation deficit” pattern and may be of diagnostic relevance for early detection and clinical follow-up.

The present results are consistent with previous findings. Rahmani et al. (2023)^14^ reported that the SVLT effectively differentiated older adults with mild cognitive impairment, Alzheimer’s disease, and normal memory performance^14^. Similarly, international studies by Alioto et al. (2017)^15^, Rabin et al. (2009)^16^, and Lee et al. (2024)^13^ confirmed that verbal learning tests showed high sensitivity in detecting early memory decline in dementia^32^. However, the results of the present study diverge from those of Aranda and Wallace (2022)^17^, who found that verbal learning tests have limited sensitivity in distinguishing mild dementia from healthy aging, recommending that such tools be used in combination with other measures^17^. Furthermore, Stricker et al. (2024)^33^ found that the CogState Brief Battery (CBB) could not differentiate participants with preclinical Alzheimer’s biomarkers from healthy individuals, suggesting that test sensitivity may depend on normative characteristics and demographic variables^33^. These discrepancies may arise from differences in sample composition, symptom severity, or cultural variations in verbal learning strategies.

Consistent with the quantitative findings presented in Table 4, a significant difference between the two groups was found only for “irrelevant responses during longdelay cued recall,” whereas other indices (repetitions, unrelated responses in immediate or free recall, and false positives in recognition) did not differ significantly. The absence of significant group differences in most error indices may indicate that, in the early or moderate stages of dementia, deficits are more pronounced in encoding and retrieval processes than in overt cognitive errors. In other words, the sensitivity of error patterns as discriminative indicators is lower at this stage. The observed difference in irrelevant responses during longdelay cued recall may reflect specific inhibitory control deficits and difficulties in distinguishing relevant from irrelevant information among dementia patients. This finding aligns with previous Iranian research by Rahmani et al. (2023)^14^ and with evidence linking frontal and executive dysfunctions to increased production of irrelevant responses in dementia^34^.

The observed associations between MMSE scores and several SVLT indices support the convergent validity of the SVLT in individuals with dementia. While the MMSE primarily functions as a brief cognitive screening and severity index, the SVLT provides a more detailed assessment of verbal learning and memory processes. The stronger associations observed for learning and free-recall measures, compared with immediate recall, emphasize the SVLT’s sensitivity to specific episodic memory mechanisms that are not fully captured by brief screening instruments such as the MMSE.

From a psychometric perspective, the findings also underscore the importance of cultural adaptation in cognitive assessment. Because the SVLT was developed using Persian-language and culturally relevant word lists, it may enhance ecological validity and diagnostic sensitivity when assessing verbal memory among Iranian older adults.

The present findings also have potential clinical implications. The ability of the SVLT to differentiate between individuals with dementia and cognitively healthy older adults suggests that it may serve as a useful tool in cognitive screening contexts, particularly for the early identification of memory impairment in older populations. In clinical practice, the SVLT may be most informative when used in conjunction with other neuropsychological measures that assess complementary cognitive domains, such as global cognition, executive functioning, and attention. Such a multimethod assessment approach may enhance diagnostic accuracy and provide a more comprehensive characterization of cognitive functioning in individuals undergoing evaluation for possible neurocognitive disorders.

These findings should be interpreted in light of several limitations. Participants were recruited using convenience and purposive sampling and were restricted to community-dwelling older women from a single geographic region (Shiraz, Iran). Although this strategy enhanced sample homogeneity, it may limit the representativeness of the sample and the generalizability of the findings to men, individuals from other regions, and more diverse cultural or clinical populations. In addition, the relatively modest sample size and cross-sectional design preclude conclusions regarding longitudinal cognitive changes. Future multicenter studies including larger and more heterogeneous samples are needed to further establish the external validity of the SVLT and to examine its predictive utility across different stages of cognitive impairment.

In conclusion, the findings indicate that the SVLT may serve as a culturally appropriate and sensitive measure for distinguishing verbal memory impairment in Persian-speaking older adults. While further validation in larger and more diverse samples is warranted, its use alongside established screening tools could contribute to earlier identification of cognitive decline in clinical and community settings.

## Data Availability

The datasets generated and/or analyzed during the current study are not publicly available due to [ethical/legal/privacy] restrictions but are available from the corresponding author upon reasonable request.
